# Comparative Proteome Analysis of *Shewanella putrefaciens* WS13 Mature Biofilm Under Cold Stress

**DOI:** 10.3389/fmicb.2020.01225

**Published:** 2020-06-09

**Authors:** Jun Yan, Jing Xie

**Affiliations:** ^1^College of Food Science & Technology, Shanghai Ocean University, Shanghai, China; ^2^Laboratory for Quality and Safety Risk Assessment of Aquatic Products in Storage and Preservation of Ministry of Agriculture and Rural Affairs, Shanghai Ocean University, Shanghai, China; ^3^Shanghai Professional Technology Service Platform on Cold Chain Equipment Performance and Energy Saving Evaluation, Shanghai Ocean University, Shanghai, China; ^4^National Experimental Teaching Demonstration Center for Food Science and Engineering, Shanghai Ocean University, Shanghai, China

**Keywords:** *Shewanella putrefaciens*, psychrotrophic bacteria, food spoilage, biofilm, cold stress, proteomics

## Abstract

Worldwide, *Shewanella putrefaciens* is the predominant seafood spoilage microorganism during cold storage. This bacterium can attach to biotic/abiotic surfaces to form biofilms which contribute to seafood quality degradation and shelf-life reduction. The mechanism of *S. putrefaciens* biofilm formation is not yet described. Crystal violet staining in combination with confocal laser scanning microscopy (CLSM) was used to study the sequence of events leading to the establishment of a mature biofilm at 4, 15, and 30°C. In addition, the main chemical constituents of the mature biofilm were determined by Raman spectroscopy (RM), whereas, comparative proteomic analysis was used to quantify changes in metabolic pathways and to find out underlying protein determinants. The physical dimensions of the mature biofilm, i.e., biomass, biovolume, and mean thickness, were higher at 4°C when compared to 15 and 30°C. The variations of proteins measured by RM confirmed the importance of proteins during the formation of a mature biofilm. Comparative proteomic analysis showed that siderophore and iron chelate transport proteins were down-regulated during mature biofilm formation. The down-regulated aforementioned proteins are involved in promoting iron storage in response to a higher demand for metabolic energy, whereas, the upregulated proteins of the sulfur relay system, pyrimidine metabolism, and purine metabolism are related to bacterial adaptability. Synthesis of proteins related to cold stress was increased and proteins involved in aminoacyl-tRNA biosynthesis were up-regulated, whereas, proteins involved in aminopeptidase activity were down-regulated. Proteolysis to scavenge energy was reduced as proteins involved in pyrophosphatase activity were up-regulated. Also extracellular eDNA was found which may play an important role in maintaining the stability of mature *S. putrefaciens* biofilm structures under cold stress. This work provides a better understanding of the role of proteins in mature biofilms. In addition, the biofilm formation mechanism of a psychrotrophic spoilage bacterial species at low temperature is explored, which may contribute to generating biofilm controlling strategies during seafood preservation and processing.

## Introduction

*Shewanella putrefaciens* is a widely distributed psychrotrophic Gram-negative bacterium (Ye et al., 2019). This Gram-negative bacterium reduces trimethylamine oxide (TMAO) to trimethylamine (Jørgensen and Huss, 1989) with concurrent production of ammonia and volatile sulfides (Gram and Melchiorsen, 1996; Ge et al., 2017) and is the predominant seafood spoilage microorganism. Adherence of *S. putrefaciens* to food processing surfaces and aquatic products leads to biofilm formation which is further prompted at low temperature (Bagge et al., 2001). Biofilm matrix protects *S. putrefaciens* cells from adverse environmental conditions, including many sanitizers which are applied on the surfaces and it represents a persistent source of contamination in the sea food industry (Yildiz and Fong, 2015).

Biofilms are complex microbial communities that adhere to a wide range of biotic and abiotic surfaces. On these surfaces, these communities are embedded in a matrix of extracellular biopolymers, such as exopolysaccharides, proteins, lipids, and extracellular DNA (Donlan and Costerton, 2002; Simões et al., 2010; Abee et al., 2011). This biofilm matrix protects bacteria from environmental stresses and makes its removal difficult. Migration of cells from biofilms in a food production facility is a food safety hazard and leads to food spoilage and economic losses due to reduced shelf-life (Simões et al., 2010; Cui et al., 2020). Shelf-life can be increased through cold storage but biofilm can still be formed under cold storage (Chmielewski and Frank, 2003). Therefore the study of biofilm formation during cold storage is relevant, practically. However, the number of studies targeting low temperature biofilm formation of psychrotrophic spoilage bacteria is not extensive.

Identifying proteins which play important roles during biofilm formation is crucial for understanding the regulation of biofilm formation as mentioned in many previous studies (Ma et al., 2009; Yildiz and Fong, 2015). Advances in “Omics” technologies have made these techniques an ideal platform for studying biofilms, providing a powerful tool to reveal the underlying mechanism involved in biofilm formation. The use of proteomics technologies to study biofilms has been demonstrated (Oosthuizen et al., 2002; Laura et al., 2019). In addition regulatory mechanisms controlling bacterial adaption to a range of environmental stresses by combining protein data with genetic information have also been elucidated (Pérez-Ibarreche et al., 2017). However, this proteomic information is lacking for describing the adaptation of *S. putrefaciens* during cold stress.

In this study, we aim to quantify the events of the *S. putrefaciens* biofilm life cycle and characterize the chemical and proteomic heterogeneity of the mature biofilm under cold stress. The results of this study contribute to a better understanding of the biofilm formation mechanism of *S. putrefaciens* under cold stress, then provide a theoretical basis for the elimination of biofilm and contribute to further control of contamination of spoilage microorganism of aquatic products during the preservation and processing.

## Materials and Methods

### Bacterial Strains and Culture Preparation

*Shewanella putrefaciens* WS 13 strain used in this study was isolated from a shrimp in a putrefactive state (*Litopenaeus vannamei*) and stored in our laboratory (Chen et al., 2019a). The strain was maintained in Luria Broth (LB, Land Bridge Technology, Beijing, China) with 50% (v/v) glycerol at −80°C. The strain was recovered in 9 mL of LB and incubated at 30°C with shaking at 200 rpm for 12 h, and repeated the same operation.

### Formation and Quantification of Biofilms

Biofilms forming assay was carried out as described previously (Zhu et al., 2019) with slight modifications. The overnight broth cultures of *S. putrefaciens* WS13 were grown to approximately 8 log CFU mL^–1^ (OD_600_≈0.8) and diluted with fresh sterile LB medium (1:100), then added 1 mL of dilution culture to 24-well polystyrene microtiter plates. Each sample was tested in six replicates. Subsequently, the samples were incubated at 4, 15, and 30°C statically to form biofilms for various time (8, 12, 24, 36, 48, 60, 72,84, 96, 108, 120, and 132 h) and the plastic wraps were used to minimize evaporative loss.

The biomass of biofilm was quantified by crystal violet staining as described previously (Stepanovic, 2000). After incubation, the supernatant was discarded. The biofilms were carefully washed three times with sterile phosphate-buffered saline (PBS, pH 7.0) to remove unattached cells, and then were fixed at 60°C for 30 min. Subsequently, biofilm was stained with 1 mL of 0.2% (w/v) crystal violet (Sangon Biotech, Co., Ltd., Shanghai, China) for 15 min at room temperature, then the wells were washed to remove the redundant dye. At last, 1 mL 33% acetic acid (v/v) (Sinopharm Chemical Reagent, Co., Ltd., Shanghai, China) was used to release the dye. The absorbance was measured by BioTek Synergy 2 (Winooski, VT, United States) at 630 nm.

### Confocal Laser Scanning Microscopy (CLSM) Analysis

After 8, 12, 24, 36, 48, 60, 72,84, 96, 108, 120, and 132 h incubation on the plastic sheet, the suspension was removed and washed by 1 mL of 0.1M PBS. Then the samples were fixed for 30 min at 4°C with 4% glutaraldehyde (Sangon Biotech, Co., Ltd., Shanghai, China), and rinsed with 0.1M PBS to remove the glutaraldehyde, then stained with SYBR Green I (Sangon Biotech, Co., Ltd., Shanghai, China) in the dark for 30 min at room temperature. The excess stain was removed using PBS then air-dried. CLSM images were acquired using the confocal laser scanning machine (LSM710, Carl Zeiss AG, Germany). A 20× microscope objective was used, the SYBR Green I was excited at 488 nm and emitted at 525 ± 25 nm. For each sample, six separate sites were acquired randomly. The CLSM images were analyzed by the ISA-2 software (Ping Chen, Shanghai Ocean University, China) to determine biofilms structural parameters including biovolume and mean thickness (Tan et al., 2018).

### Extraction of EPS

The extraction of the EPS was performed using the probe sonication extraction protocols (Gong et al., 2009; Qiao et al., 2017). The broth cultures of *S. putrefaciens* WS13 was diluted at 1:100 ratio with fresh sterile LB medium, and dilutions were transferred into individual wells of 6-well plates (5 mL/well). The plates were statically incubated at 30, 15, and 4°C for 24, 72, and 84 h, respectively. Then the suspended cultures were discarded and washed with 5 mL of 0.1M PBS to remove loose suspended cells. Subsequently, the biofilm samples were suspended in a 5 mL of 0.01 M KCl by vortexing and scraping then harvested. The cells were disrupted with a sonicator (VCX 500, SONICS, Newtown, CT, United States) for four cycles of 5 s of operation and 5 s of pause at a power level of 3.5 Hz. The sonicated suspension was centrifuged for 20 min (4,000 rcf, 4°C), and the suspension was then filtered through a membrane filter (0.22 μm) to ensure cell-free, EPS suspension.

### Raman Spectroscopy Analysis

The composition of EPS was analyzed by using the Raman spectra with a Senterra R200-L Dispersive Raman Microscope (Bruker Optics, Ettlingen, Germany) at room temperature. A 50× microscope objective was used, and the sample was excited using 45–50 mW of a 785 nm diode laser. The Raman signal was collected in the spectral interval 400–1,400 cm^–1^. To record biofilm Raman spectra, spectra were collected at 15 points for each sample. Spectrum analysis and preprocessing of preliminary data were carried out using the Bruker OPUS software (Tan et al., 2018).

### Proteomic Analyses

#### Protein Digestion

Protein concentrations were measured using the Bicinchoinci acid (BCA) method by a BCA Assay Kit (Thermo Fisher Scientific, United States). Each sample tube contained 150 μg of protein. The sample solution was added to [*tris*(2-carboxyethyl) phosphine] (TCEP) to reach a final concentration of 10 mM and incubated at 37°C for 60 min. Then, an appropriate quantity of iodoacetamide (IAM) was added to achieve a final concentration of 40 mM and incubated for 40 min in the dark. Finally, 100 m Mtriethylammonium bicarbonate (TEAB) buffer was added to dilute the concentration of the solution. Trypsin solution, in the ratio 1:50, was then added to each sample tube and incubated at 37°C overnight (Shi et al., 2018).

#### Mass Spectrometry Analysis and Protein Identification

The samples (nine samples from three groups) were analyzed on a Q Exactive mass spectrometer coupled to an Easy-nLC 1200nano-flow UHPLC. Each sample was loaded onto the C18-reversed phase column (75 μm × 25 cm, Thermo Fisher Scientific, United States) having two solvent systems (buffer A: 2% acetonitrile and 0.1% formic acid; buffer B: 80% acetonitrile and 0.1% formic acid) for 160 min at a flow rate of 300 nL/min. The full scan MS spectra ranged from 350 to 1300 m/z and were acquired with a mass resolution of 70 K.

MS/MS spectra were screened by Proteome Discoverer^TM^ 2.2 software (Thermo Fisher Scientific, United States) against the *S. putrefaciens* (31682 entries) from UniprotKB. The highest score for a given peptide mass (best match to that predicted in the database) was used to identify parent proteins. Tryptic digestion with up to two missed cleavages, carbamidomethylation of cysteines as a fixed modification, and oxidation of methionines and protein N-terminal acetylation as variable modifications. A 1% false discovery rate (FDR) was used to identify peptide spectral matches based on *q*-values.

### Statistical and Bioinformatic Analysis

All experiments were tested at least in triplicate on different occasions. The data were expressed as the mean ± standard deviation. Correlation analysis was analyzed by the Pearson method using the software of Statistical Product and Service Solutions (SPSS, Inc., Chicago, IL, United States). Differences at *p*-value < 0.05 were considered statistically significant. Differential expression analysis on the proteins identified at three temperatures was performed. 4°C was used as the experimental group, 15 and 30°C were the control group. Student’s *t*-test with a *p*-value of 0.05 and proteins showing at least 1.2 fold changes were considered for further analysis. We focused on the up-regulated protein intersection and down-regulated protein intersection in 4 vs. 15°C and 4 vs. 30°C samples. GO^1^ enrichment analysis was carried out to define the functional categories of these proteins which were repeatedly confirmed as important for low-temperature status of *S. putrefaciens.* KEGG^2^ enrichment analysis was carried out to investigate possible acting pathways of these proteins. Furthermore, proteins enriched significantly in KEGG pathway were selected for global protein interaction network analysis using STRING^3^ and Cytoscape tools.

## Results

### Biofilm Development

**FIGURE 1:**
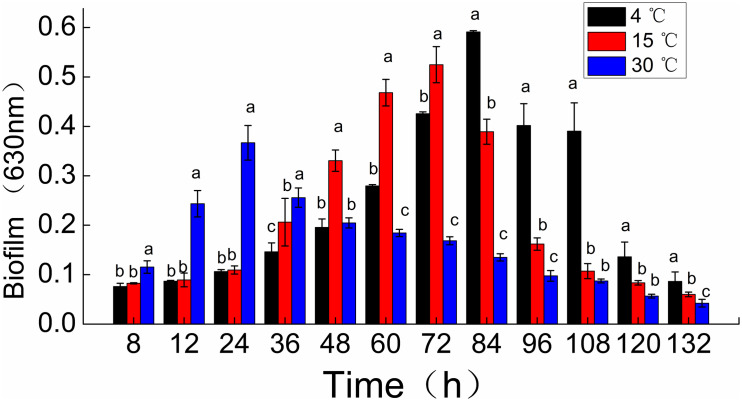
Time course of *Shewanella putrefaciens* WS13 biofilm production for 8,12, 24, 36, 48, 60, 72, 84, 96, 108, 120, and 132 h at 4, 15, and 30°C. Biofilm biomass (OD_630_ nm) by crystal violet staining method. The error bar represents the standard deviation of triplicate experiments. *a, b, c means in the same column with different superscripts are significantly different (*p* < 0.05).

*Shewanella putrefaciens* WS13 biofilm formation was observed dynamically using crystal violet staining (Figure 1). The peak amount of biomass in biofilms of *S. putrefaciens* WS13, as measured by OD at 630 nm, was 0.367, 0.525, and 0.591. These peak biomass amounts occurred at 24, 72, and 84 h after incubation at 30, 15, and 4°C. The rate of biofilm formation was therefore quickest during incubation temperature at 30°C when compared to incubation 4 and 15°C. However, the total amount of biomass formed at 132 h during incubation at 4 and 15°C for was much higher compared to that at 30°C.

### Confocal Laser Scanning Microscopy Imaging

**FIGURE 2:**
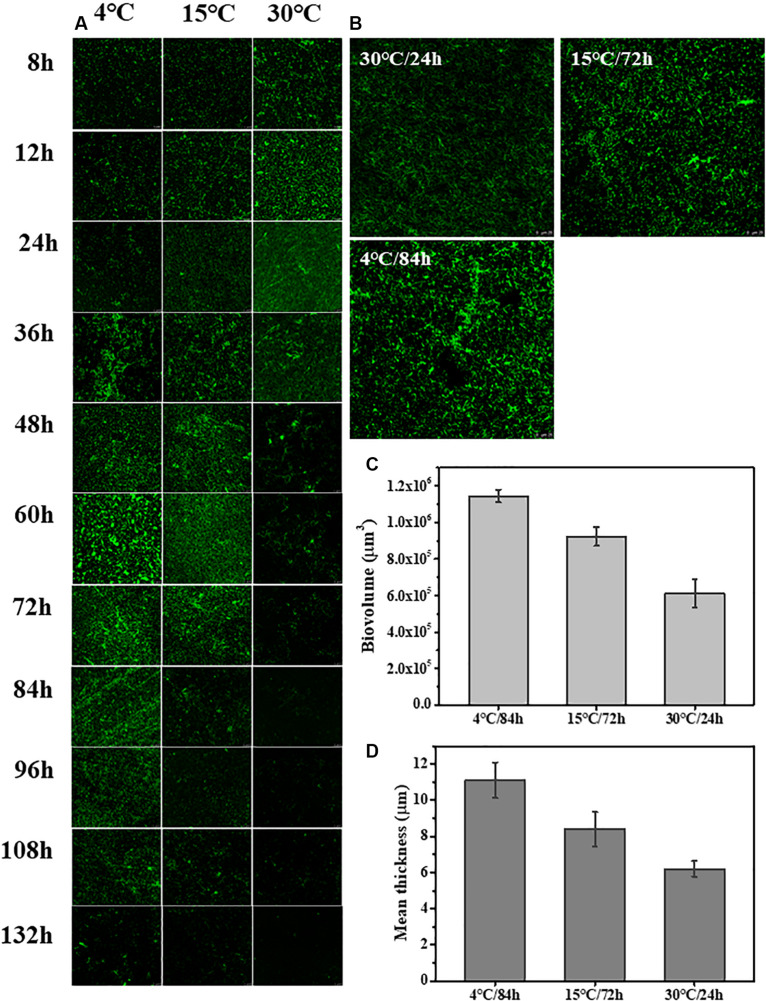
Time course of *S. putrefaciens* WS13 biofilm production for 8, 12, 24, 36, 48, 60, 72, 84, 96, 108, 120, and 132 h at 4, 15, and 30°C. The images were representative of three independent replicates. **(A)** Representative CLSM images of biofilm formed by *S. putrefaciens* WS13 after 8, 12, 24, 36, 48, 60, 72, 84, 96, 108, 120, and 132 h incubation. **(B)** Representative CLSM images of the mature biofilm formed by *S. putrefaciens* WS13 at 4, 15, and 30°C. **(C)** The biovolume of the mature biofilm at different temperature. **(D)** The mean thickness of the mature biofilm at different temperature. The error bar represents the standard deviation of triplicate experiments.

The changes to the morphological structure of the biofilm over 132 h were measured using CLSM (Figure 2). The CLSM analysis followed a similar trajectory with the crystal violet staining measurements. Biovolume and mean thickness of the biofilm, calculated from the CLSM images, were used as indexes for quantifying morphology and structural characteristics. The experimental surface was covered by a mature biofilm after 84 h when incubated 4°C with a biovolume of (11.4 ± 0.35) × 10^5^ μm^3^ and mean thickness of 11.1 ± 0.98 μm. In contrast, the experimental surface was covered by a mature biofilm after only 24 h when incubated at 30°C with a biovolume of (6.1 ± 0.79) × 10^5^ μm^3^ and mean thickness of 6.2 ± 0.42 μm. At 15°C, the experimental surface was covered by the mature biofilm at 72 h with a biovolume of (9.2 ± 0.52) × 10^5^ μm^3^ and mean thickness of 8.4 ± 0.95 μm.

### Analysis of EPS in a Mature Biofilm

**FIGURE 3:**
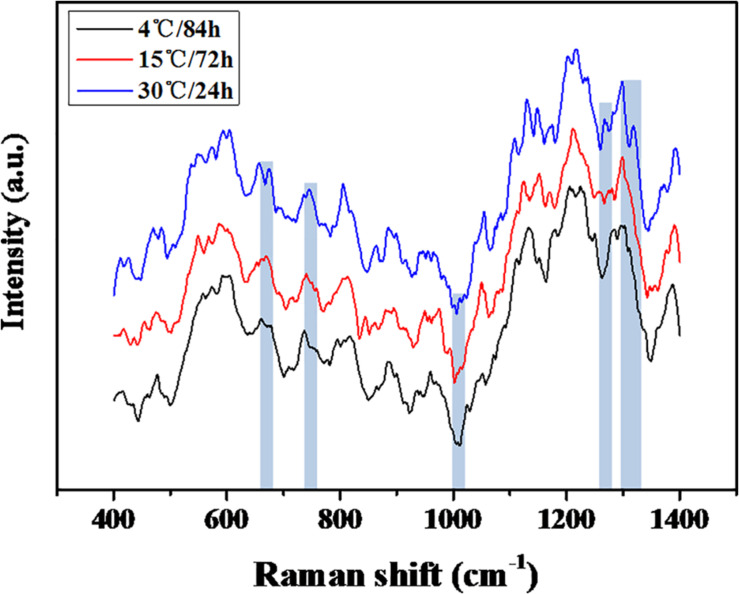
Raman spectra of the mature biofilm of the *S. putrefaciens* W13.

The quantification of the chemical composition of the biofilm was monitored in the range 400 to 1,400 cm^–1^ (Figure 3). The tentative peak assignments of the Raman bands are summarized in Table 1. Typical vibrational bands of carbohydrates, proteins, and nucleic acid were shown in the Raman spectra of biofilm. Protein accounted for four significant Raman EPS bands: 637–695, 1000–1010, 1235–1260, and 1300 cm^–1^. The peak at 788 cm^–1^ indicated an O-P-O stretch of DNA which existed widely in nucleic acid. The Raman spectroscopy showed that the protein content in the biofilms changes significantly, indicating the important role of protein in maintaining the structural stability of biofilms (Yildiz and Fong, 2015; Taglialegna et al., 2016). And the variation of nucleic acid also indicated the content of nucleic acids may also be an important factor to maintain the stability of the mature biofilm which is consistent with the previous study (Jakubovics et al., 2013; Okshevsky and Meyer, 2015).

**TABLE 1:** Assignment of Raman bands of EPS in biofilms.

Wave number (cm^–1^)	Assignment	Macromolecular assignment	References
637–695	C–S str and C–C twisting of proteins (tyrosine)	Proteins	Ivleva et al., 2009; Ramya et al., 2010; Chen et al., 2019b
782–788	O-P-O stretch of DNA	Nucleic acids	Samek et al., 2014; Qiao et al., 2017
1000–1010	C–C aromatic ring stretching (phenylalanine)	Proteins	Guicheteau et al., 2008; Ivleva et al., 2008; Kahraman et al., 2009
1235–1260	Amide III	Proteins	Laucks et al., 2005; Ivleva et al., 2008; Jarvis and Goodacre, 2008
1300	Skeletal benzene stretches and N-C pyrrole stretching	Proteins	Bleich et al., 2015

### Protein Identification

**FIGURE 4:**
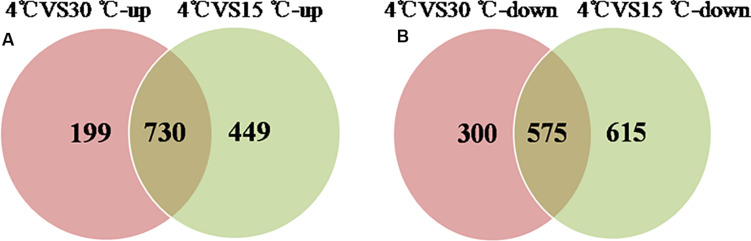
Venn-diagram of numbers of differential proteins. **(A)** The number of up-regulated proteins of the mature biofilm at 4°C compared to 15 and 30°C. **(B)** The number of down-regulated proteins of the mature biofilm at 4°C compared to 15 and 30°C.

Comparative proteomic analyses were used to identify differentially expressed proteins during the formation of a mature biofilm cultured at 4°C (Figure 4) and compared to mature biofilms cultured at 15 and 30°C. A total of 1378 proteins were classified as up-regulated and 730 were simultaneously up-regulated. And 1490 proteins were identified as down-regulated, of which 575 were simultaneously down-regulated. These differentially expressed proteins play an important role in the development of the biofilm under cold stress (*p* < 0.05, at least 1.2-fold change).

GO functional enrichment analysis was performed to gain insights into the functions of these differentially expressed proteins. All proteins were grouped according to their participation in biological process (BP), building cellular component (CC), and molecular function (MF). The GO function of the top 20 differentially expressed proteins was analyzed further.

**FIGURE 5:**
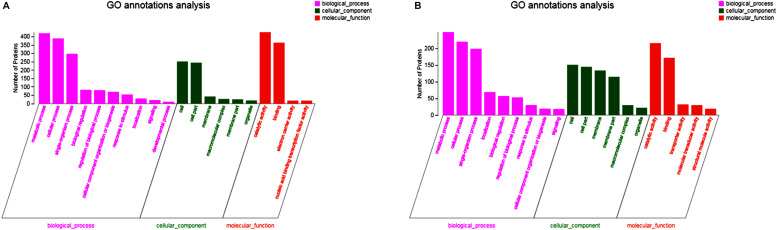
GO functional analysis of differential expressed proteins. **(A)** The GO functional of up-regulated proteins of the mature biofilm at 4°C compared to 15 and 30°C. **(B)** The GO functional of down-regulated proteins of the mature biofilm at 4°C compared to 15 and 30°C.

**FIGURE 6:**
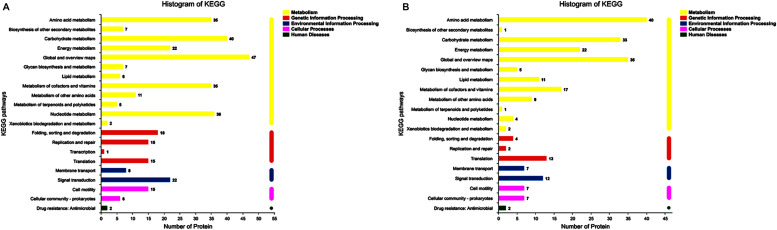
KEGG annotation of differential expressed proteins. **(A)** The KEGG analysis of up-regulated proteins of the mature biofilm at 4°C compared to 15 and 30°C. **(B)** The KEGG analysis of down-regulated proteins of the mature biofilm at 4°C compared to 15 and 30°C.

In up-regulated proteins, proteins involved in metabolic, cellular, single-organism process were prominent in BP. In addition, the cell and cell part were the two most abundant categories in CC and catalytic activity and binding were significant in MF (Figure 5A and Supplementary Table S1). Further, KEGG analysis indicated that these proteins were primarily involved in the pathway of the sulfur relay system, aminoacyl-tRNA biosynthesis, pyrimidine metabolism, RNA degradation, methane metabolism, and purine metabolism (Figure 6A and Supplementary Table S2). The individual pathways with enriched up-regulated proteins are provided in Supplementary Figure S1.

In down-regulated proteins, proteins involved in metabolic, cellular, single-organism process were also prominent in BP, while cell, cell part, membrane, membrane part were the most abundant categories in CC. Proteins involved in catalytic activity and binding were also significant in MF (Figure 5B and Supplementary Table S3). KEGG analysis showed that these downregulated proteins were primarily involved in the pathway of tyrosine metabolism, oxidative phosphorylation, valine, leucine and isoleucine degradation, propanoate metabolism, and phenylalanine metabolism (Figure 6B and Supplementary Table S4). The individual pathways with enriched down-regulated proteins are provided in Supplementary Figure S2.

### Interaction Network of Differentially Expressed Proteins

Proteins interact with each other to form a variety of functional connections. These include stable complexes, metabolic pathways, and an array of direct and indirect regulatory interactions. The interaction of the differentially expressed proteins identified in this study was analyzed using STRING to derive protein interaction networks during low temperature biofilm metabolism. A total of 88 up-regulated proteins were significantly enriched or co-expressed in the KEGG pathway and 77 of these proteins were identified after removing the redundant ones. Among the down-regulated proteins, a total of 43 were significantly enriched in the KEGG pathway, and 36 down-regulated differential proteins were obtained by removing the redundant ones. The final protein interaction network analysis diagram was constructed by using 77 up-regulated proteins and 36 down-regulated proteins.

**FIGURE 7:**
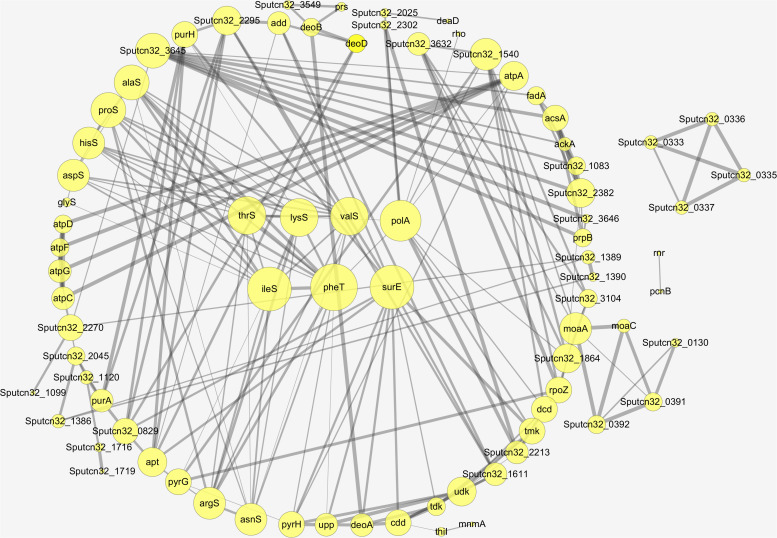
The protein–protein interaction network of the differentially expressed proteins.

The nodes in the figure represent proteins and the size of the node indicates the intensity of the interaction. In addition thickness of the connecting line shows the degree of association discovered by STRING (Figure 7). There were seven proteins that had the most interaction with other proteins, A4Y6G9, A4Y4A4, A4Y938, A4YCC9, E6XLB9, A4Y9 × 9, A4Y928 as shown in the middle part of Figure 7 (see also Table 2). The results indicate that the proteins involved in Aminoacyl-tRNA biosynthesis and nucleic acid metabolism play a significant role in low temperature biofilm formation of *S. putrefaciens*.

**TABLE 2:** The function description and pathway definition of the interacting protein.

Identifier		Description	Pathway_definition
pheT	A4Y6G9	Phenylalanine-tRNA ligase beta subunit	Aminoacyl-tRNA biosynthesis
ileS	A4Y4A4	Isoleucine–tRNA ligase	Aminoacyl-tRNA biosynthesis
surE	A4Y938	5′-Nucleotidase SurE	Nicotinate and nicotinamide metabolism, pyrimidine metabolism, purine metabolism
polA	A4YCC9	DNA polymerase I	Base excision repair, DNA replication, homologous recombination, nucleotide excision repair, pyrimidine metabolism, purine metabolism
thrS	E6XLB9	Threonine-tRNA ligase	Aminoacyl-tRNA biosynthesis
lysS	A4Y9 × 9	Lysine-tRNA ligase	Aminoacyl-tRNA biosynthesis
valS	A4Y928	Valine-tRNA ligase	Aminoacyl-tRNA biosynthesis

## Discussion

*Shewanella putrefaciens* exhibits broad temperature adaptability. In the cold chain, this microorganism exists as biofilms and is responsible for the majority of spoilage of seafood and seafood products. The mechanism underlying biofilm formation of *S. putrefaciens* under cold stress is only now being studied. For this purpose, phenotypic traits for biofilm formation at 4, 15, and 30°C were investigated initially. These temperatures were chosen to mimic optimal growth at 30°C, typical cold chain temperature at 4°C and an intermediate temperature of 15°C.

In this study, the biomass of the mature biofilm of *S. putrefaciens* W13 at 4 and 15°C was much higher than that at 30°C. Phenotypically, the concurrent increase of biovolume and mean thickness of a biofilm was similar to mature biofilms produced by other psychrotrophic bacteria. This is a common trait among biofilm producing psychrotrophic bacteria where low temperature promotes the coordinated expression of genes and proteins to strengthen the architectural and structural integrity of biofilms (Chierici et al., 2016).

The heterogeneity of the chemical components of mature biofilms formed at different temperatures were quantified using RM (Figure 3). The chemical components of the mature biofilms formed at different temperatures were quite different, and a significant variation in protein contents was observed, indicated proteins played an important role in maintaining the stability of mature biofilms under cold stress (Yildiz and Fong, 2015; Taglialegna et al., 2016). In addition, the variation in the types of nucleic acid also indicates that the content of nucleic acids may also be an important factor for the mature biofilm (Jakubovics et al., 2013; Okshevsky and Meyer, 2015).

Proteomic analysis is a powerful technique for investigating protein expression patterns and has been used to study the growth of psychrotrophic bacteria. An adaptation mechanism for surviving in a low temperature environment is the availability of iron. At 4°C, the proteins responsible for siderophore transport and iron chelate transport were exclusively down-regulated during the growth of *S. putrefaciens* W13. A number of authors have documented that iron was important for robust biofilm formation and that the iron concentration was a 100-fold higher than what was needed for normal growth (Branda et al., 2001; Weinberg, 2004; Banin et al., 2005). It suggests that storing iron by bacterial cells is an adaptive response in the face of higher demands of metabolic energy (Dhungana et al., 2003) and necessity for maturing biofilms.

In maturing biofilms of *S. putrefaciens* subjected to cold stress, proteins involved in Aminoacyl-tRNA biosynthesis were up-regulated and proteins involved in aminopeptidase activity were down-regulated. This adaptive response indicates that protein synthesis rather than protein degradation was optimized to maintain the stability of the mature biofilm. In addition, proteins involved in pyrophosphatase activity were up-regulated to compensate for increased energy requirements of a cell under cold stress as energy derived from proteolysis was reduced.

Proteins involved in the sulfur relay system, pyrimidine metabolism, purine metabolism were up-regulated, indicating that under cold stress the formation of biofilm caused significant changes in bacterial physiological metabolism. The significantly enriched KEGG pathways by upregulated proteins at 4°C versus 15 and 30°C showed that pyrimidine metabolism, purine metabolism, and RNA degradation pathway were the most obvious enrichment (Supplementary Figure S1 and Supplementary Table S2). That indicated the nucleic acid metabolism was active. eDNA is also one of the main components of the biofilm matrix and mainly derived from the lysis of some dead bacteria cells in the biofilm. eDNA could help the biofilm gradually develop from colony stage to maturity, and the interaction between eDNA and exopolysaccharides was conducive to the formation of a more complex “mushroom”-like three-dimensional structure (Dominik et al., 2011; DeLeo et al., 2014). eDNA is required to maintain the structural integrity of biofilms and the important role of eDNA in maintaining structural stability has been demonstrated by using DNase treatment (Okshevsky and Meyer, 2015; Sadiq et al., 2019). The proteins involved in helicase activity were up-regulated and would facilitate the nucleic acid metabolism and maintain the stable structure of biofilm. According to the previous study (Rowland et al., 2011), the induction of helicase transcript was one of the earliest and largest transcriptional responses to cold, the helicase encoded is critical for cold acclimation.

In conclusion, this study characterized the heterogeneity of proteins involved in the formation of a mature biofilm under different temperatures, and confirmed the important roles of these particular proteins in maintaining the structural stability of the mature biofilm under cold stress. However, the signaling pathways and subsequent regulation of protein expressions remain to be studied. Overall, the results of this study can provide references for a more comprehensive study into the regulatory networks and biochemical pathways in the mature biofilm development process of the *Shewanella* spp.

## Conclusion

A pioneering proteome profile of a mature biofilm formed by *S. putrefaciens* under different temperatures is presented in this work. This study extends the knowledge of the physiology of *S. putrefaciens* WS 13 growing in a biofilm mode of survival under cold stress. Proteomic results were consistent with microbiological ones where low temperatures promote an increase in biofilm biomass and increases in a range of protein determinants related to biofilm formation. The mechanism information provided by this study can inform strategies for reducing the impact of this economically important spoilage microorganism.

## Data Availability Statement

The mass spectrometry proteomics data have been deposited to the ProteomeXchange Consortium (http://proteomecentral.proteomexchange.org/cgi/GetDataset?ID=PXD019333) and the iProX (https://www.iprox.org/page/project.html?id=IPX0002203000).

## Author Contributions

JY and JX designed the research. JY wrote the manuscript. JX revised the manuscript.

## Conflict of Interest

The authors declare that the research was conducted in the absence of any commercial or financial relationships that could be construed as a potential conflict of interest.

## Footnotes

1 http://www.geneontology.org/

2 http://www.kegg.jp/

3 https://string-db.org/
