# Supplementary material for: Comparative Proteome Analysis of Shewanella putrefaciens WS13 Mature Biofilm Under Cold Stress
Source: Front Microbiol. 2020 Jun 9;11:1225. doi: 10.3389/fmicb.2020.01225 (PMC7296144; doi:10.3389/fmicb.2020.01225)

Figure S1. This file shows the significantly enriched KEGG pathways by upregulated 730 proteins in 4 ^o^C S.*putrefaciens* versus 15 and 30 ^o^C. Deep green: protein enriched to the pathway; light green: species enriched to the pathway; red ring or blue ring: protein set.

(A). Pathway_ID : spc04122. KEGG Description: Sulfur relay system


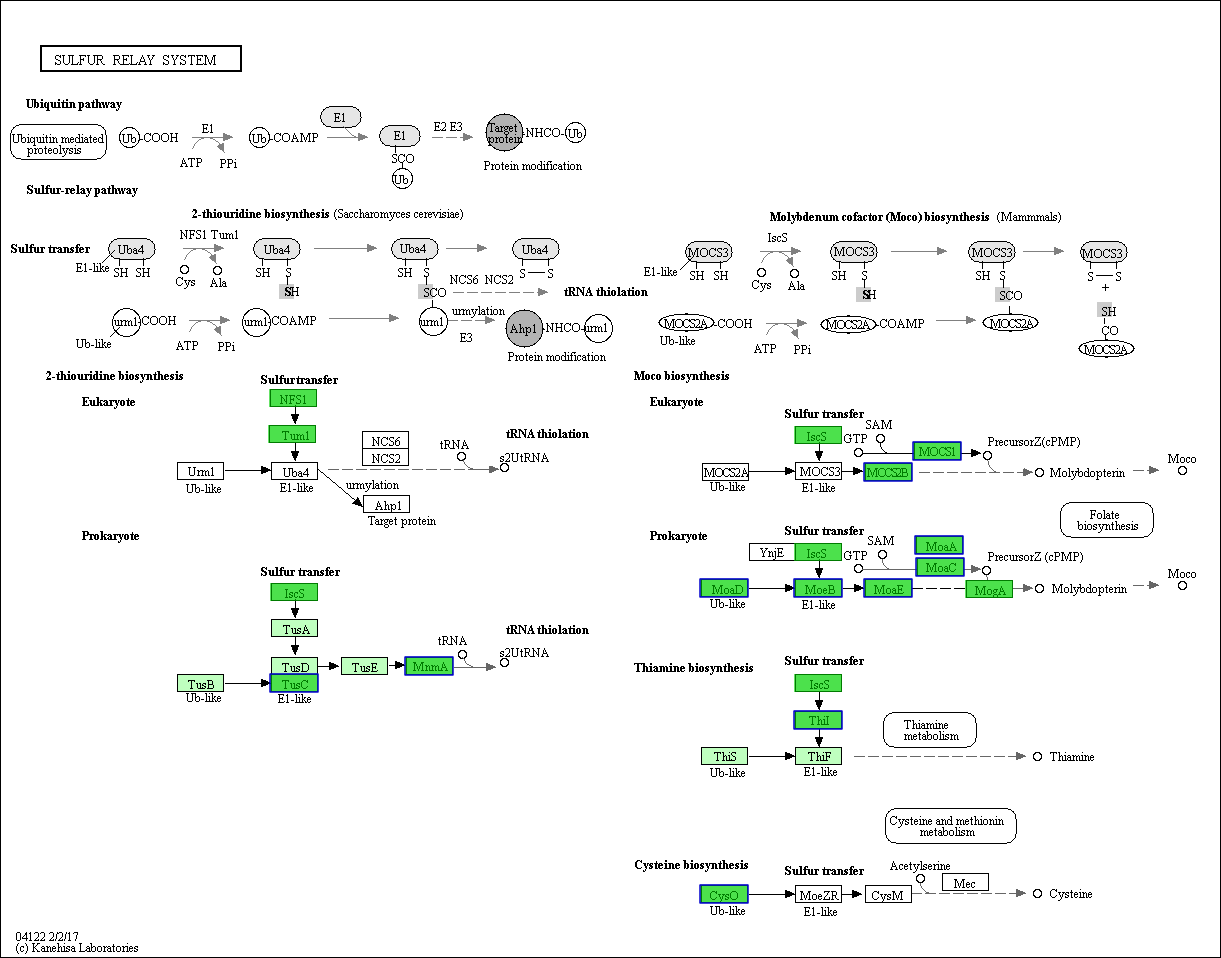


(B) Pathway_ID : spc00970. KEGG Description: Aminoacyl-tRNA biosynthesis


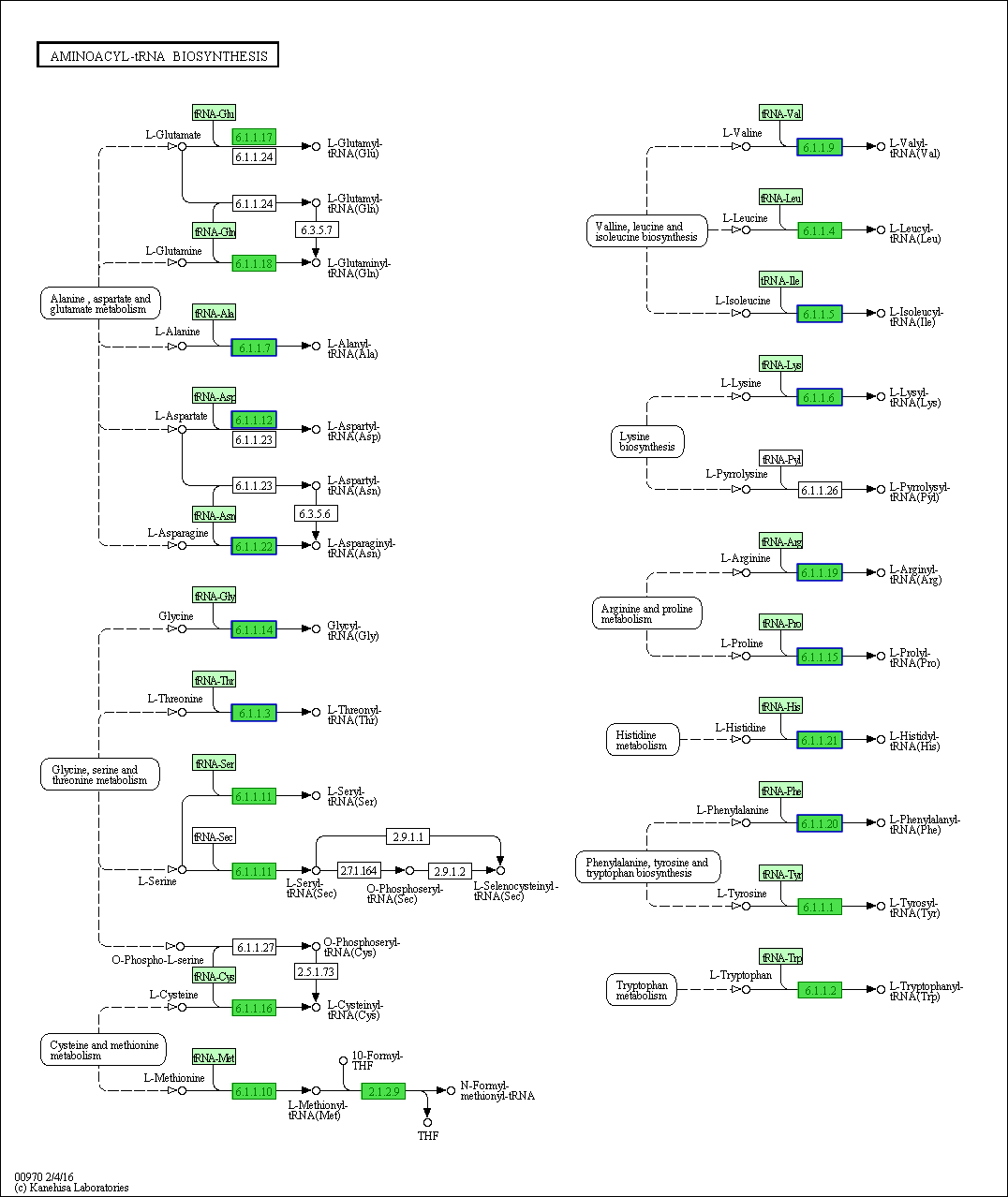


(C) Pathway_ID : spc00240. KEGG Description: Pyrimidine metabolism


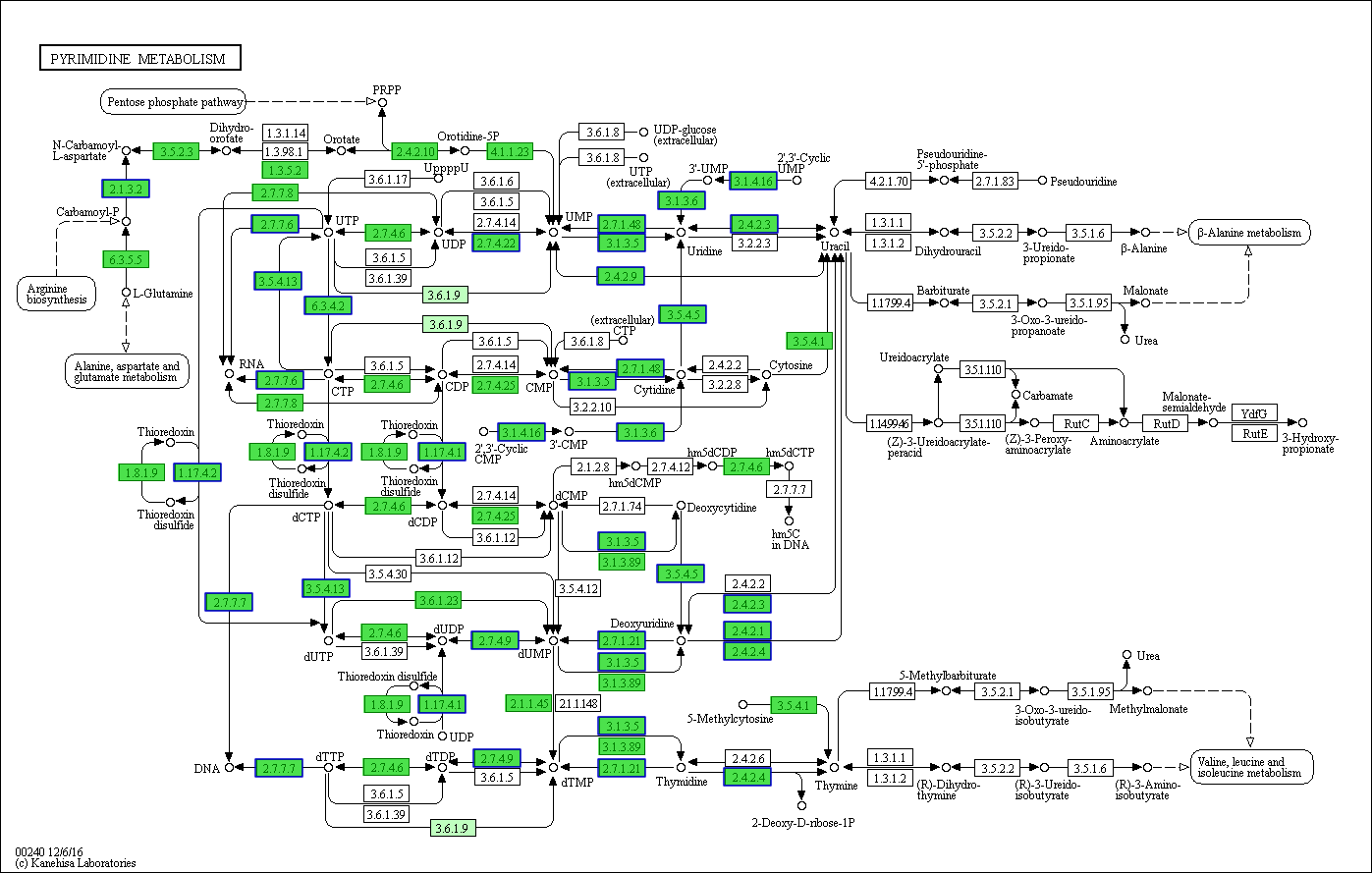


(D) Pathway_ID : spc03018. KEGG Description: RNA degradation


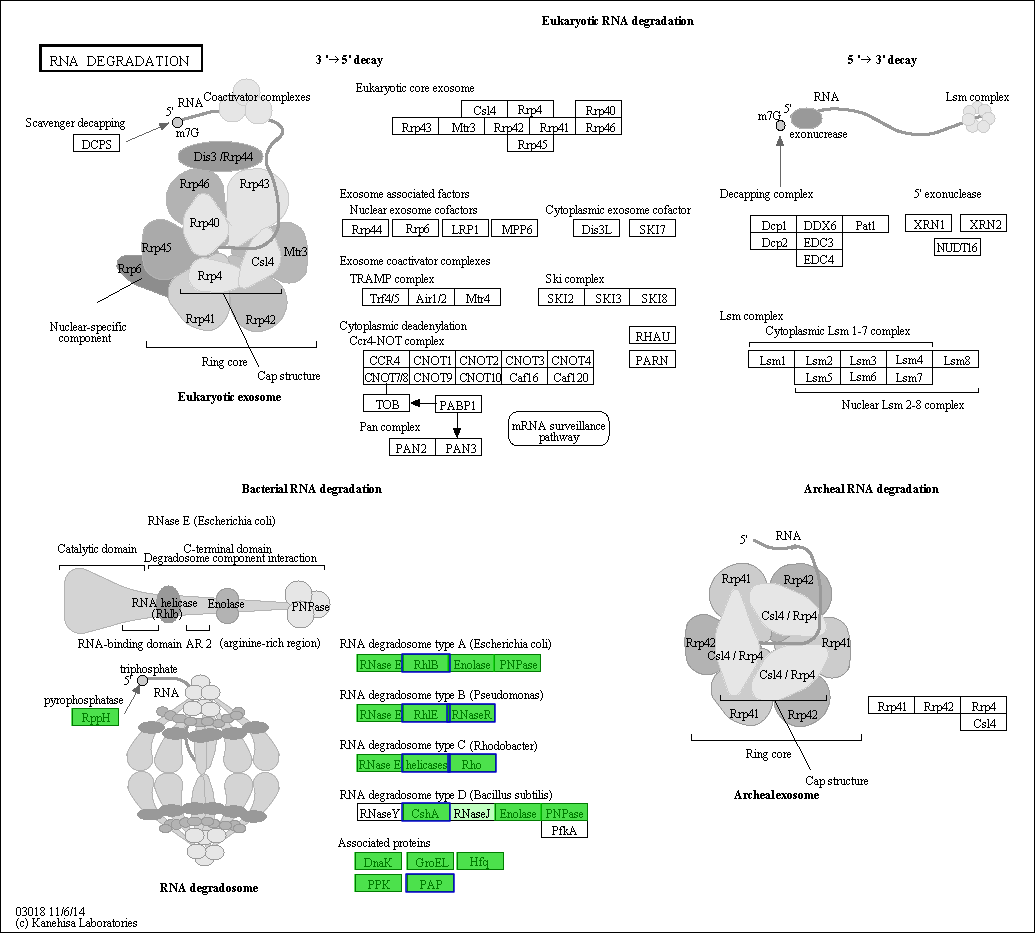


(E) Pathway_ID : spc00680. KEGG Description: Methane metabolism


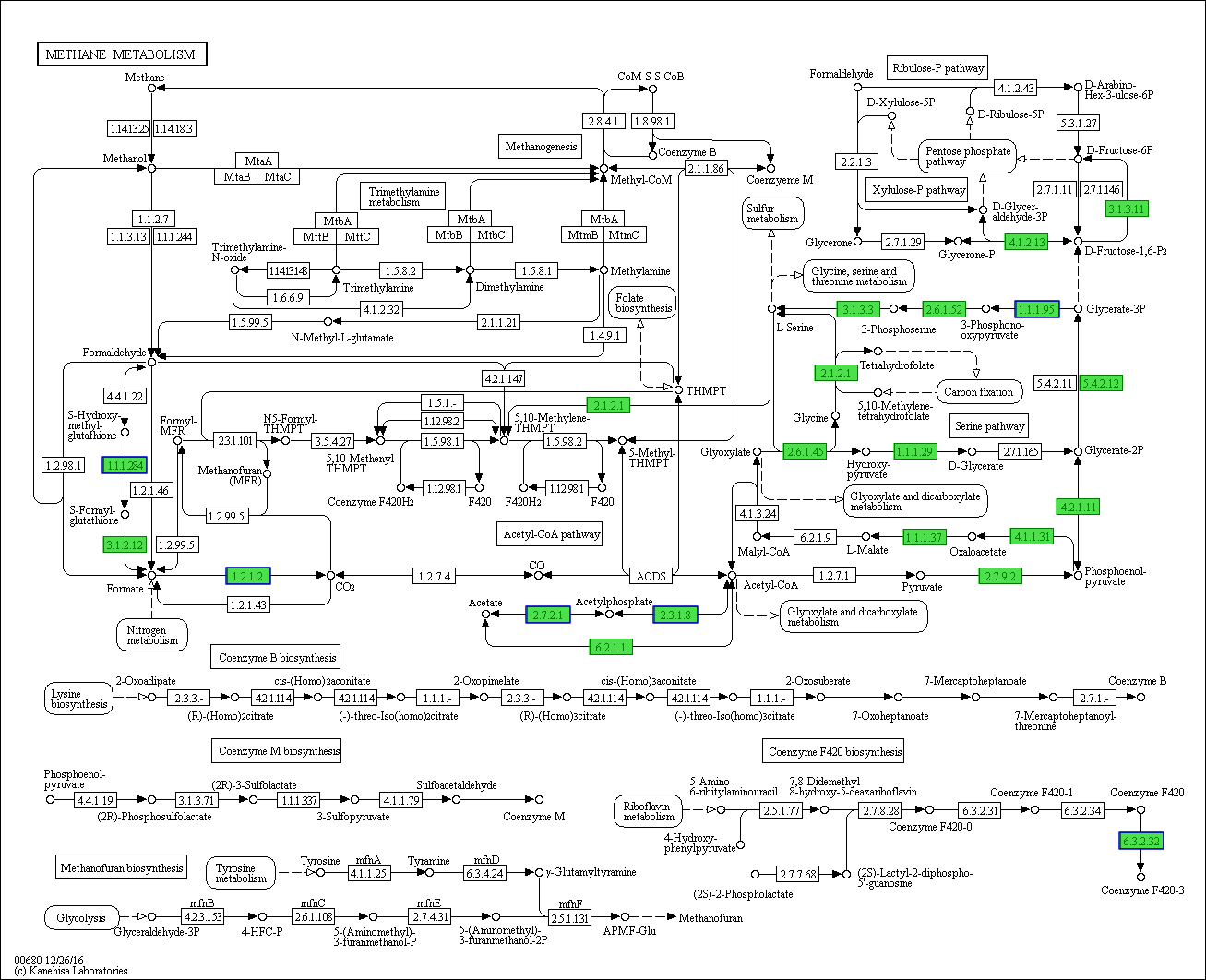


(F) Pathway_ID : spc00230. KEGG Description: Purine metabolism


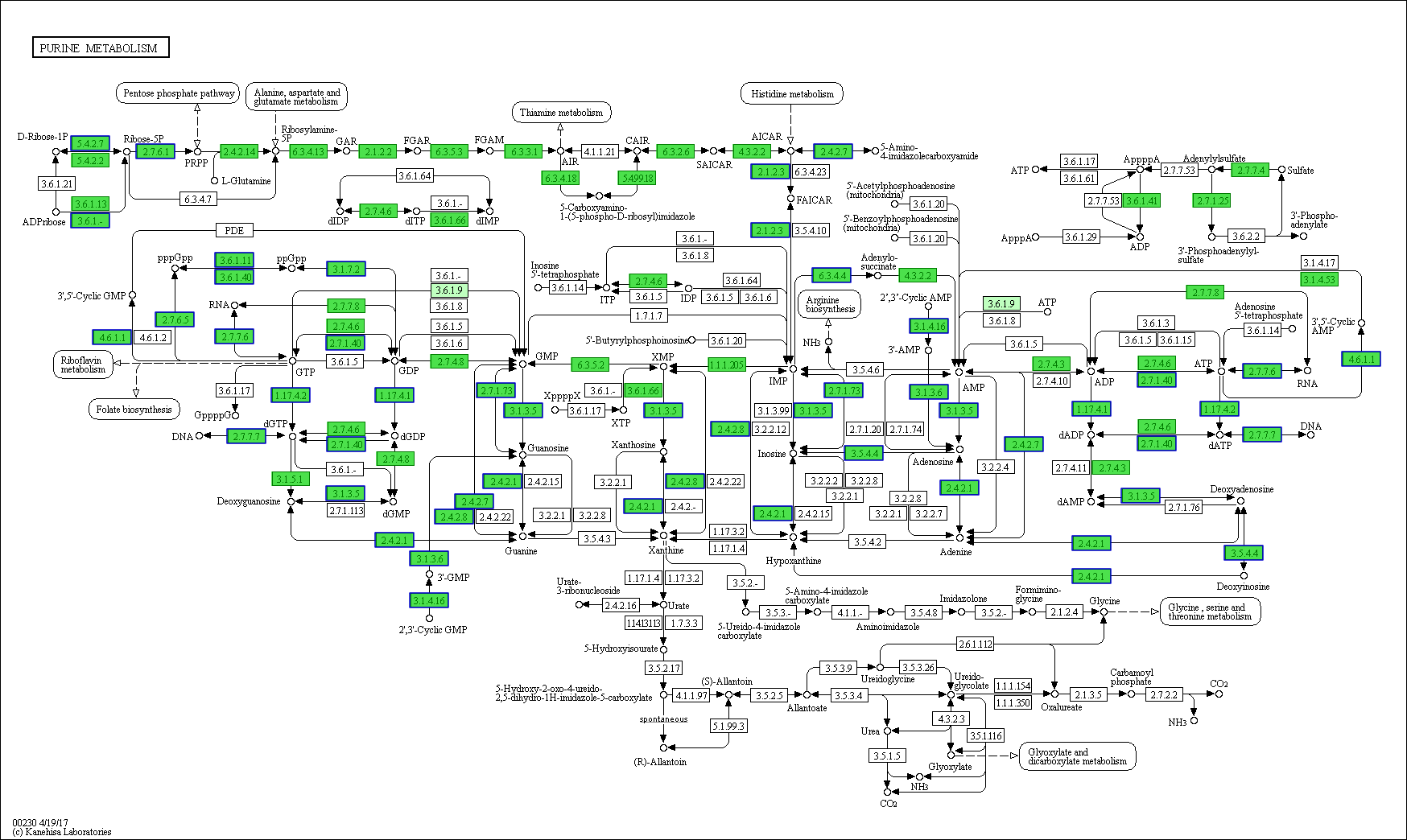

Supplement: Supplementary file 1 [file Data_Sheet_1.zip › Figure S1.DOCX]
