# Supplementary material for: Comparative Proteome Analysis of Shewanella putrefaciens WS13 Mature Biofilm Under Cold Stress
Source: Front Microbiol. 2020 Jun 9;11:1225. doi: 10.3389/fmicb.2020.01225 (PMC7296144; doi:10.3389/fmicb.2020.01225)

Figure S2. This file shows the significantly enriched KEGG pathways by downregulated 535 proteins in 4 ^o^C *S. putrefaciens* versus 15 and 30 ^o^C. Deep green: protein enriched to the pathway; light green: species enriched to the pathway; red ring or blue ring: protein set.

(A) Pathway ID: spc 00280. KEGG Description: Tyrosine metabolism


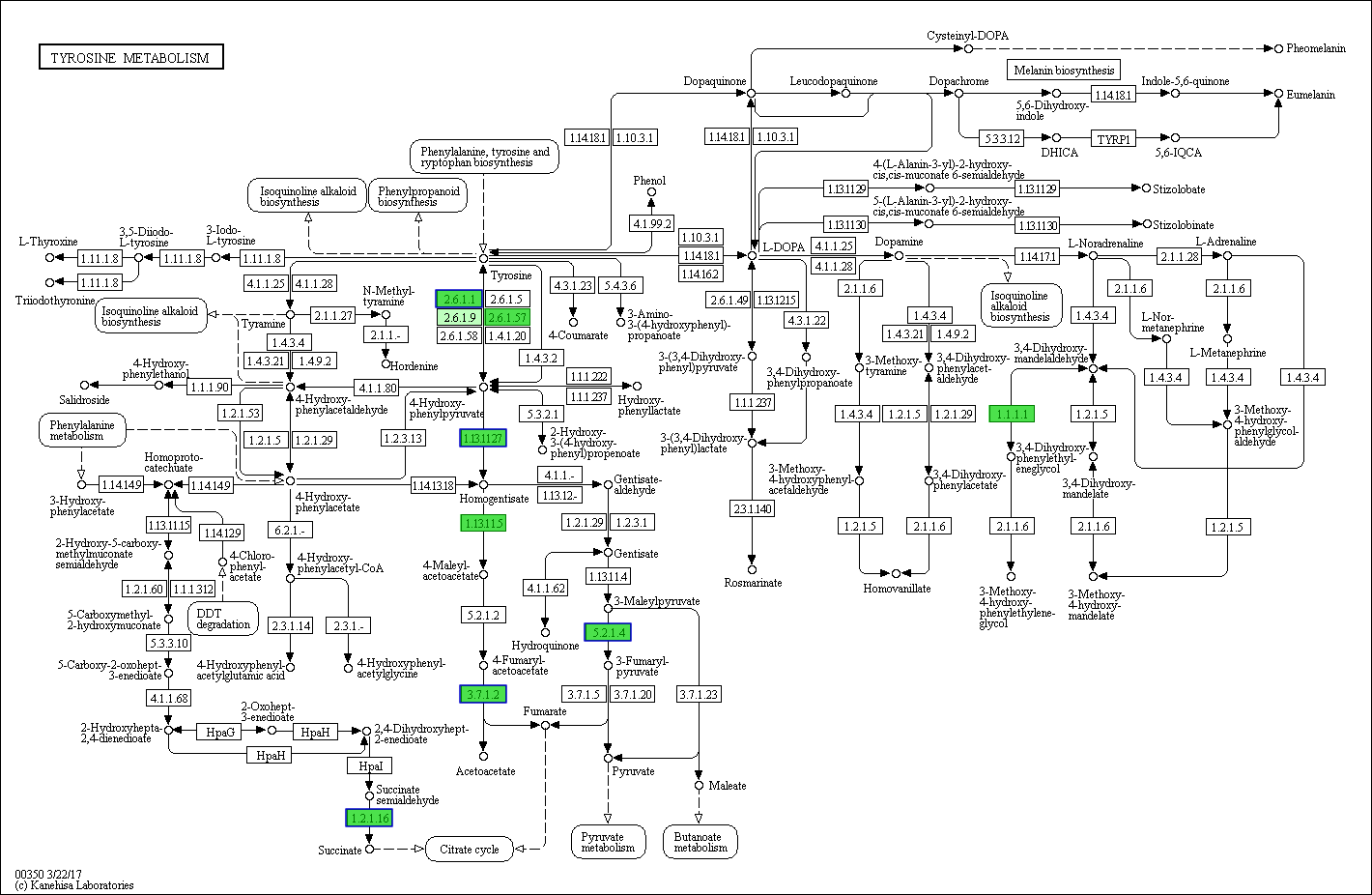


(B) Pathway ID: spc 00280. KEGG Description: Oxidative phosphorylation


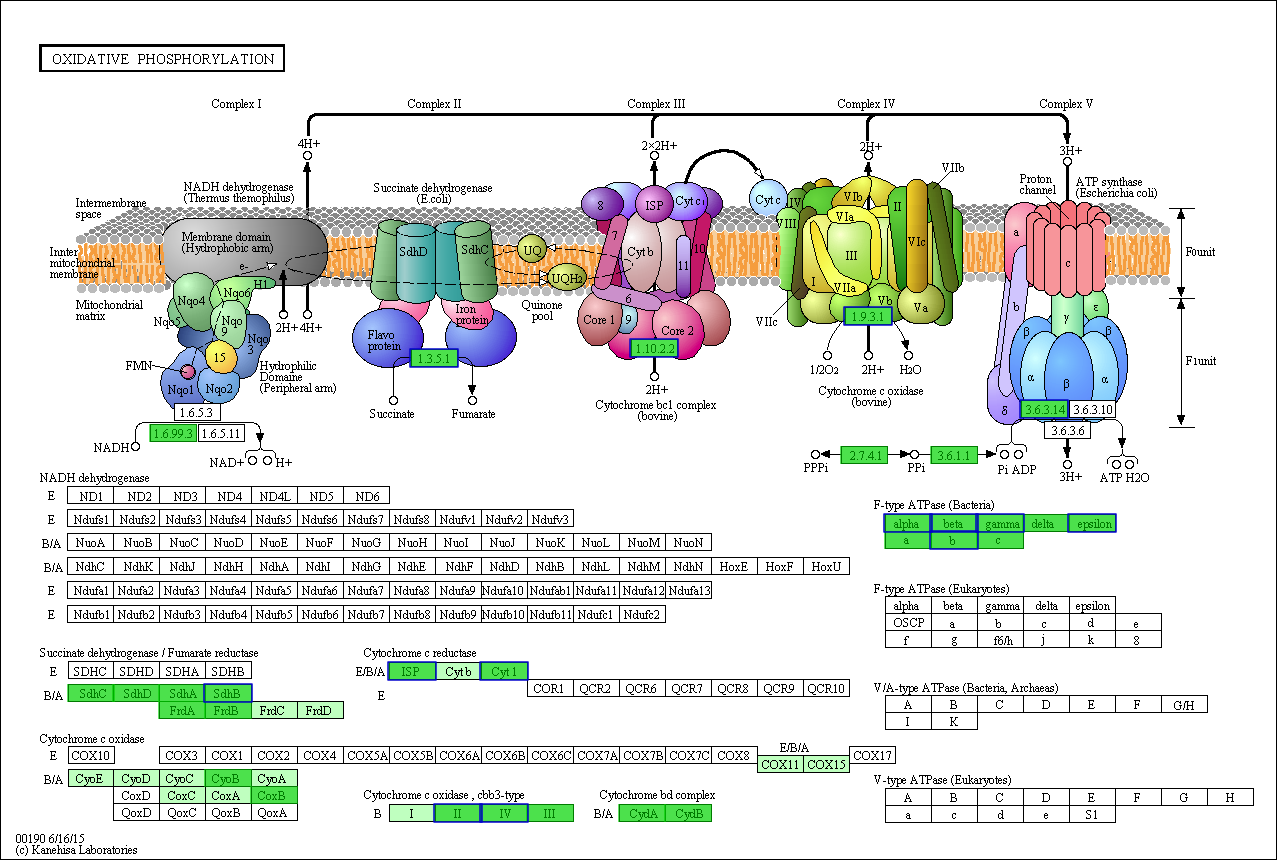


(C) Pathway ID: spc 00280. KEGG Description: Valine leucine and isoleucine degradation


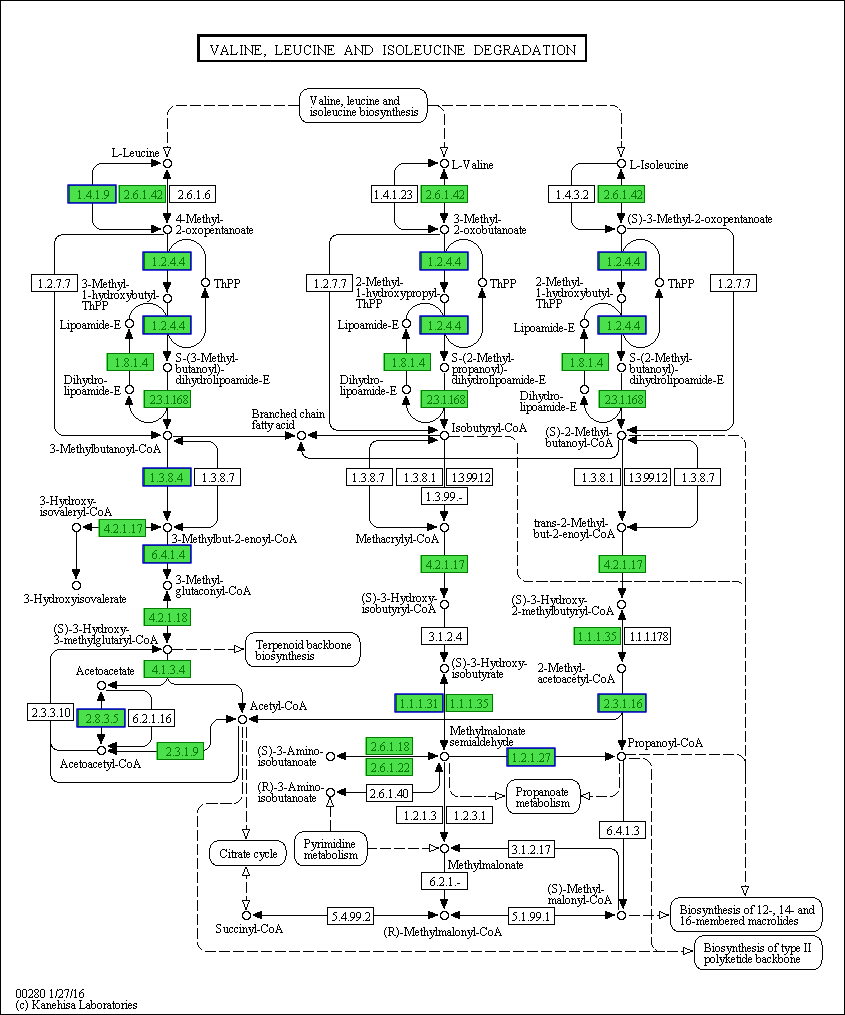


(D) Pathway ID: spc 00640. KEGG Description: Propanoate metabolism


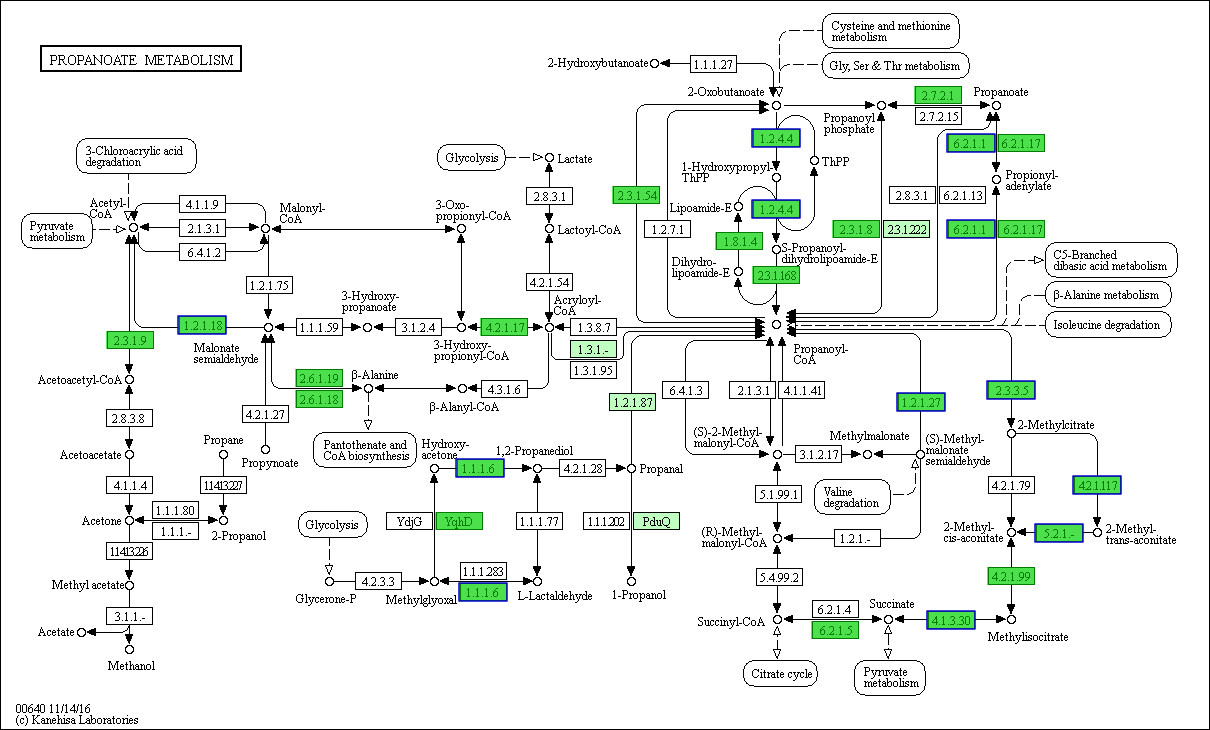


(E) Pathway ID: spc 00360. KEGG Description: Phenylalanine metabolism


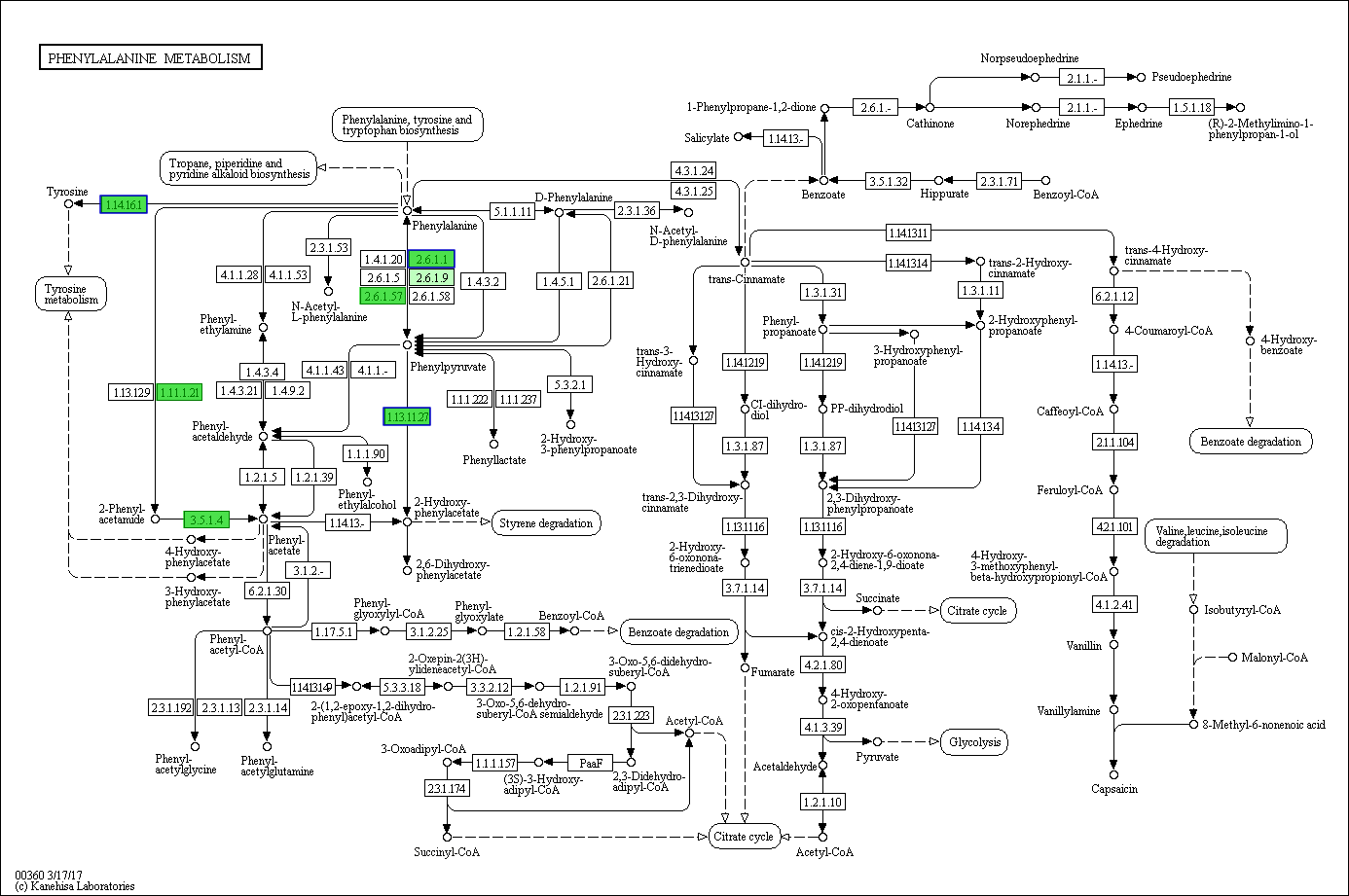

Supplement: Supplementary file 1 [file Data_Sheet_1.zip › Figure S2.DOCX]
