# Supplementary material for: Comparative Proteome Analysis of Shewanella putrefaciens WS13 Mature Biofilm Under Cold Stress
Source: Front Microbiol. 2020 Jun 9;11:1225. doi: 10.3389/fmicb.2020.01225 (PMC7296144; doi:10.3389/fmicb.2020.01225)
Supplement: Supplementary file 1 [file Data_Sheet_1.zip › Supplementary Tables.DOCX]

**Supplementary table 1. The top 20 GO function of the up-regulated proteins**

| **Protein Number** | **GO ID** | **GO Description** | ***P*-value uncorrected** |
| --- | --- | --- | --- |
| 23 | GO:0004386 | helicase activity | 1.28983E-06 |
| 71 | GO:0016462 | pyrophosphatase activity | 2.12282E-06 |
| 70 | GO:0017111 | nucleoside-triphosphatase activity | 2.23283E-06 |
| 71 | GO:0016818 | hydrolase activity, acting on acid anhydrides, in phosphorus-containing anhydrides | 2.28519E-06 |
| 71 | GO:0016817 | hydrolase activity, acting on acid anhydrides | 2.28519E-06 |
| 146 | GO:0035639 | purine ribonucleoside triphosphate binding | 2.45463E-06 |
| 146 | GO:0001883 | purine nucleoside binding | 2.45463E-06 |
| 146 | GO:0032550 | purine ribonucleoside binding | 2.45463E-06 |
| 147 | GO:0017076 | purine nucleotide binding | 2.77922E-06 |
| 122 | GO:0005524 | ATP binding | 2.92427E-06 |
| 122 | GO:0032559 | adenyl ribonucleotide binding | 2.92427E-06 |
| 208 | GO:0006725 | cellular aromatic compound metabolic process | 3.05771E-06 |
| 122 | GO:0030554 | adenyl nucleotide binding | 3.15735E-06 |
| 54 | GO:0034660 | ncRNA metabolic process ncRNA | 3.22372E-06 |
| 173 | GO:0005737 | cytoplasm | 3.38864E-06 |
| 170 | GO:0043168 | anion binding | 3.39715E-06 |
| 159 | GO:0097367 | carbohydrate derivative binding | 3.53447E-06 |
| 193 | GO:0000166 | nucleotide binding | 3.62733E-06 |
| 193 | GO:1901265 | nucleoside phosphate binding | 3.62733E-06 |
| 146 | GO:0001882 | nucleoside binding | 3.67177E-06 |

**Supplementary table 2.The KEGG analysis of the up-regulated proteins**

| **Pathway ID** | **Description** | **Proteinnumbers** | **Proteins** |
| --- | --- | --- | --- |
| spc00680 | Methane metabolism | 11 | A4Y282;  E6XM93;  E6XQG6;  A0A379ZTJ3;  A4Y285;  E6XIY3;  A4Y284;  E6XLJ6;  A4Y286;  E6XIJ0;  A4Y820 |
| spc00240 | Pyrimidine metabolism | 22 | A4Y9A7  E6XRF2  A0A252EZ97  A0A379ZN97  A4Y7K6  A4Y938  A4Y7K5  E6XIE8  E6XJ71  A4YCC9  E6XJX9  A4Y7K1  A4Y5V3  A4Y4G5  A4Y5N2  E6XKK5  E6XGL0  E6XP05  E6XR72  A0A252EWL3  A4Y7U0  E6XN94 |
| spc00230 | Purine metabolism | 25 | E6XKE8  A4Y3M6  A4YAX9  A4Y938  A0A366IWL9  A4YBK7  A4Y3E4  A4YBC5  E6XJ71  A4YCC9  E6XJX9  A4Y7K1  A4YA34  A4Y5V3  A4Y9A6  E6XP05  E6XKK5  A4Y5N2  A0A252EWL3  E6XP06  E6XQ20  A4Y6K4  A4Y7U0  A4Y7T3  A4Y983 |
| spc04122 | Sulfur relay system | 8 | A4Y1N3  A4Y2D8  A4Y4X5  A4Y6Z1  A4Y7M1  A4Y2E1  A4Y2E0  A0A366IRM7 |
| spc03018 | RNA degradation | 9 | A4YA59  A4Y713  E6XM63  A4YB09  A4Y334  A0A366IT17  A4Y3E6  A4YA38  A0A448ERF6 |
| spc00970 | Aminoacyl-tRNA biosynthesis | 13 | E6XNJ4  A4Y9X9  E6XLB9  A4Y4A4  A4Y6E4  E6XID6  A4Y2U9  A4Y6S5  A4Y931  E6XHH6  A4Y6G9  A4Y8F6  A4Y928 |

**Supplementary table 3. The top 20 GO function of the down-regulated proteins**

| **Protein Number** | **GO ID** | **GO Description** | ***P*-value uncorrected** |
| --- | --- | --- | --- |
| 12 | GO:0015891 | siderophore transport | 7.9636E-08 |
| 12 | GO:0015688 | iron chelate transport | 7.9636E-08 |
| 32 | GO:0044462 | external encapsulating structure part | 4.04632E-07 |
| 15 | GO:1901678 | iron coordination entity transport | 6.27595E-07 |
| 31 | GO:0009279 | cell outer membrane | 1.08154E-06 |
| 34 | GO:0019867 | outer membrane | 1.35668E-06 |
| 25 | GO:0005506 | iron ion binding | 1.70645E-06 |
| 127 | GO:0016020 | membrane | 3.19861E-06 |
| 115 | GO:0044425 | membrane part | 7.33519E-06 |
| 30 | GO:0060089 | molecular transducer activity | 1.35433E-05 |
| 6 | GO:0008239 | dipeptidyl-peptidase activity | 1.74274E-05 |
| 6 | GO:0070009 | serine-type aminopeptidase activity | 1.74274E-05 |
| 107 | GO:0016021 | integral component of membrane | 2.32263E-05 |
| 107 | GO:0031224 | intrinsic component of membrane | 2.3895E-05 |
| 29 | GO:0004872 | receptor activity | 2.67328E-05 |
| 12 | GO:0051649 | establishment of localization in cell | 0.00011581 |
| 19 | GO:0017171 | serine hydrolase activity | 0.000351056 |
| 19 | GO:0008236 | serine-type peptidase activity | 0.000351056 |
| 16 | GO:0008238 | exopeptidase activity | 0.000463293 |
| 5 | GO:0036442 | hydrogen-exporting ATPase activity | 0.000565841 |

**Supplementary table 4. The KEGG analysis of the down-regulated proteins**

| **Pathway ID** | **Description** | **Proteinnumbers** | **Proteins** |
| --- | --- | --- | --- |
| spc00360 | Phenylalanine metabolism | 4 | A0A366ISP3;  A4Y580;  E6XI44;  A4Y733 |
| spc00350 | Tyrosine metabolism | 7 | A0A366ISP3;  A4Y584;  A4Y4E4;  E6XNM3;  A4Y583;  E6XI44;  A4Y733; |
| spc00280 | Valine, leucine and isoleucine degradation | 10 | E6XNB8;  A0A366IPQ8;  A0A252EQD1;  E6XRX7;  A4Y658;  E6XRX3;  A4Y4C8;  A4Y661;  E6XN36;  E6XM43;  E6XK19; |
| spc00640 | Propanoate metabolism | 11 | A4YBM0;  E6XRH1;  A4Y7Y7;  E6XRX3;  A4Y4C8;  A0A380B1D9;  A4YBM1;  E6XRH0;  E6XN36;  E6XM43;  E6XK19 |
| spc00190 | Oxidative phosphorylation | 11 | E6XLF9;  A4YCH8;  E6XNG7;  A0A366IWA0;  E6XL00;  A4Y6U7;  E6XL02;  A4Y7Q8;  A4YCH7;  E6XNG5;  E6XKZ9 |
